# Identification of nutritional components in unripe and ripe *Docynia delavayi (*Franch.) Schneid fruit by widely targeted metabolomics

**DOI:** 10.7717/peerj.14441

**Published:** 2022-12-12

**Authors:** Can Chen, Xi Xia, Dawei Wang

**Affiliations:** 1 Southwest Forestry University, Key Laboratory for Forest Resource Conservation and Utilization in the Southwest Mountains of China, Kunming, China; 2 Southwest Forestry University, Key Laboratory for Forest Genetic and Tree Improvement & Propagation in Universities of Yunnan Province, Kunming, China

**Keywords:** *Docynia delavayi* (Franch), Metabolites, PCA, OPLS-DA, UPLC-MS/MS, KEGG

## Abstract

*Docynia delavayi* (Franch.) Schneid is an evergreen tree with multiple benefits and high development and utilization value. The fruit is consumed as fresh and dry fruit, juices, and other products. However, it is unknown the chemical changes that occur upon fruit maturation. The metabolite content of unripe and ripe fruit was examined using UPLC-MS/MS technology based on a broadly targeted metabolome. We identified 477 metabolites, of which 130 differed between ripe and unripe fruit. These compounds are primarily involved in the biosynthesis of secondary metabolites, such as pantothenic acid, flavonoids, and amino acids. Moreover, in ripe fruit, there are 94 metabolites that are upregulated, particularly flavonoids and terpenoids. In comparison, compounds associated with sour flavors (amino acids, phenolic acids, organic acids) are down-regulated. Remarkably, these metabolites have a strong relationship with the medicinal properties of *D. delavayi*. This study provides a global perspective of the *D. delavayi* fruit metabolome and a comprehensive analysis of metabolomic variations during fruit development, thereby increasing the knowledge of the metabolic basis of important fruit quality traits in *D. delavayi* fruit.

## Introduction

*Docynia delavayi* (Franch.) Schneid (Rosacea), is widely known as Duo-Yi in China. In some provinces like Yunnan, Guizhou, and Sichuan, its planting concentrates in the valleys, creeks, and shrubs at an altitude ranging between 1,000 and 3,000 m (Ci et al., 2020). The flowering season of *D. delavayi* spans from March to April of the year, and the fruiting season lasts from September to December of the year (Su, 2019). *D. delavayi* as a sort of fruit has a distinctive taste, showing high nutritional and medicinal value. In southwest China, it is commonly applied for the treatment of digestive disorders and hypertension (Xukun et al., 2014). Fruit extracts are rich in secondary metabolites and natural active compounds, including flavonoids, dietary fiber, amino acids, terpenoids, and alkaloids (Mei et al., 2002). In light of the above, *D. delavayi* fruit immaculously satisfies the functional fruit requirements, thus is considered a desirable fruit.

As a crucial part of natural growth for plants, the development and ripening of fruit are not only regulated by internal genetic information but also affected by external factors (Ramos-Aguilar et al., 2021). During the course of ripening, the variations in sweetness, hardness, and colour are the significant influencing factors for fruit quality (Bapat et al., 2009). Thus, the research of fruit growth and ripening is needed to improve our understanding as to the formation of fruit mass in *D. delavayi*. Despite some research that has already been conducted on the fruit composition of *D. delavayi* (Lee, Mattheis & Rudell, 2012), it is focused mainly on some particular metabolites. Unripe *D. delavayi* and ripe *D. delavayi* differ in their nutrient metabolite profiles. For this reason, to better understand the difference between them, a systematic analysis is required to understand the changes in composition.

Metabolomics can be applied to identify and analyze the metabolites of fruit, which is essential for determining its nutritional value (Quinet et al., 2019; Xu et al., 2021). Broadly targeted metabolomics combines the benefits of both untargeted and targeted metabolomics. Due to its high efficiency, high sensitivity, and extensive coverage, targeted metabolomics has already been widely applied to the study of metabolic changes in various fruits (Sawada et al., 2009; Chen et al., 2013). Based on ultra-performance liquid chromatography/tandem mass spectrometry (UPLC-MS/MS), metabolomics has a library for the easy recognition of various compounds. It has already been adopted to analyze metabolite profiles and to examine the variations in plant composition (Xiao et al., 2014; Wang et al., 2017; Li et al., 2021). For instance, metabolomics analysis can be conducted to examine the metabolite changes in those apples subjected to various ripening treatments (Lee, Mattheis & Rudell, 2012). The metabolomics method can be used to decode the metabolic changes in strawberry during the course of development and ripening (Zhang et al., 2010).

This study is purpose to determine the metabolic differences between unripe *D. delavayi* and ripe *D. delavayi* by applying a targeted metabolomics strategy. Such metabolites as amino acids, phenolic acids, organic acids, flavonoids and terpenoids, all of which contribute to increasing nutritional value, are the focus of the present study. The findings of it could help improve our understanding as to the metabolism of key mass characteristics in fruit, thus providing a solution to the planting of high-quality *D. delavayi*.

## Materials and Processes

### Sample collection

On July 18, 2020, the samples were collected from Lancang country (100°21′E, 22°56′N) in Yunnan province, China. Field experiments were approved by the National Natural Science Foundation of China (project number: 32060350) and the samples were collected with the consent of Liu Yu and Zhang Xinluo villagers; Nuozadu village committee for supporting this study. Immediately afterwards, the harvested fruits were transported to a laboratory, where they were graded by maturity and colouring stage: green unripe (GW) and red ripe (GX). (Su, 2019). Approximately 15 fruits in each stage were combined to represent one biological replicate, with three biological replicates prepared for each stage. All samples were frozen with dry ice.

### Sample preparation and extraction

These biological samples were freeze-dried by using a vacuum freeze-dryer (Scientz-100F). Then, the freeze-dried samples were crushed for 1.5 min at 30 Hz by a mixer mill (MM 400, Retsch GmbH, Haan, Germany) fitted with a zirconia bead. 100 mg of lyophilized powder was dissolved in 1.2 mL of 70% methanol solution, and then stirred for 30 s at a 30-minute interval (6 times in total). Finally, the samples were placed in a refrigerator at 4 °C overnight. After 10 min of centrifugation at 12,000 rpm, the extracts were filtrated (SCAA-104, 0.22 µm pore size; ANPEL, Shanghai, China) for subsequent UPLC-MS/MS analysis.

### UPLC-MS/MS conditions

Given the limited availability of extracts, the application of ultra-performance liquid chromatography coupled to tandem mass spectrometry is considered to be one of the preferred methods of analysis. The following UPLC conditions are required for the present study (Chen et al., 2013):

1. Column, Agilent SB-C18 (1.8 µm, 2.1 mm*100 mm);
2. The mobile phase included solvent A:
  a. MilliQ water with 0.1% formic acid, and solvent B:
  a. Acetonitrile with 0.1% formic acid.

### ESI-Q TRAP-MS/MS

**Figure 1:**
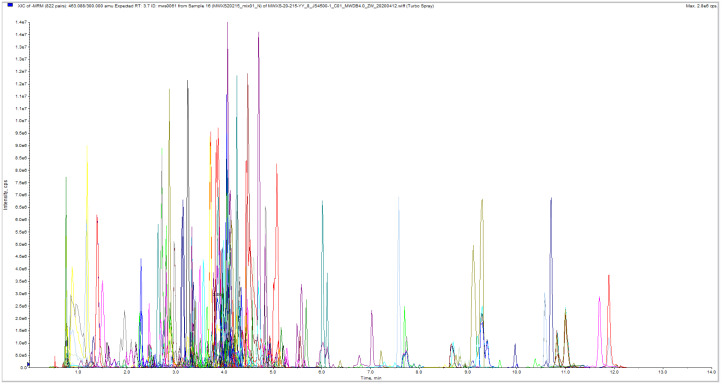
The multi-peak map of MRM metabolite detection.

An AB4500 Q TRAP UPLC/MS/MS system, complete with an ESI Turbo Ion-Spray interface, was adopted for both LIT and Triple Quadrupole (QQQ) scanning. This system was operated by using Analyst 1.6.3 software (AB Sciex). The operational settings for the ESI source are as follows: ion source, turbo spray; source frequency of 550 °C; ion spray voltage (IS) 5,500 V (positive ion mode)/−4,500 V (negative ion mode); ion source gases I (GSI), II (GSII), and curtain gas (CUR) fixed at 50, 60, and 25.0 psi, respectively; and large collision-activated dissociation (CAD) value. To tune the device and calibrate the weight, polypropylene glycol liquids of 10 and 100 mol/L were used, respectively, in the QQQ and LIT modes. During MRM investigation, QQQ scans were obtained with the collision gas (nitrogen) adjusted to medium. Both DP and CE were further enhanced to support single MRM conversions. The MRM transitions for each session were tracked by eluting the metabolites during each interval (Fig. 1) (Fraga et al., 2010).

### Metabolite identification and quantification

MetWare databases were used. Match scores were given by using the MetDNA software developed by Zhu et al. (2013) and the Masterview software developed by AB Sciex (Toronto, Canada). The majority of the compounds in the database are standards. MRM information was collected from each standard (Fig. 1). For metabolite quantification, multiple reaction monitoring was performed. In the MRM mode, the quadrupole filtered the precursor ions of the target substance first, excluding the ions corresponding to other molecular weight substances for the preliminary elimination of interference. After the collision chamber was induced to ionize, the precursor ions were fragmented to obtain many fragment ions. Then, triple quadrupole filtering was conducted, with a distinctive fragment ion used to remove non-target ion interference for improving the accuracy of quantification and reproducibility. The peak area of all substance mass peaks was integrated after the data on metabolite mass spectrometry was collected from several samples. Besides, the peaks of the same metabolite in various samples were integrated and corrected.

### Metabolite data analysis

Principal Component Analysis (PCA) was carried out with the statistically functional prcomp in R (http://www.r-project.org). The data was unit variance scaled before unsupervised PCA. The heatmaps of dendrograms were used to present the sample and the hierarchical clustering results of metabolites. The correlation function in R can be used to calculate the Pearson correlation coefficients between samples, which are displayed in the form of heatmaps. The differential metabolites designated among groups were identified by VIP >= 1 and absolute Log_2_FC (fold change) >= 1. Obtained from OPLS-DA outcome, VIP values include two plots of score and permutation as formed with R package Metabo Analyst R. The statistic was log transfer (log2) and mean centering before OPLS-DA. A permutation test (200 permutations) was performed to stop overfitting. Pathway database (http://www.kegg.jp/kegg/pathway.html) was used to make explanation using the KEGG Compound database (http://www.kegg.jp/kegg/compound/). Then, metabolite sets enrichment analysis was conducted, and the significance of pathways with heavily regulated metabolites was determined according to the *p*-values of the hypergeometric test.

### Statistical analysis

Through Excel (Microsoft Excel 2016, Microsoft, Redmond, WA, USA), a statistical analysis was carried out. The statistics are presented as means ± standard deviations. The least significant difference (*p* < 0.05) was used to assess the level of statistical significance.

## Results

### Widely targeted metabolome profiling of *D. delavayi* fruit

To better understand the variation in nutritional values between unripe *D. delavayi* and ripe *D. delavayi*, ‘GW’ and ’GX’ were applied for the metabolic profiling based on UPLCMS/MS. With a total of 727 distinct metabolites identified in all samples, they were classified into 11 categories according to metabolite structure, as shown in Table 1. A significant number of these metabolites are suspected to be responsible for fruit development (53 amino acids and derivatives, 72 phenolic acids, 12 tannins, 29 organic acids). Among these 11 compounds, phenolic acids (72 types) and flavonoids (111 types) were considerably more abundant than the others.

**Table 1:** Classification of the detected metabolites in D. delavayi fruits into major classes.

Class	Number of Compounds
Amino acids and derivatives	53
Phenolic acids	72
Nucleotides and derivatives	33
Flavonoids	111
Lignans and Coumarins	18
Others	46
Tannins	12
Alkaloids	18
Terpenoids	24
Organic acids	29
Lipids	61

### Multivariate analyses of determined metabolites

**Figure 2:**
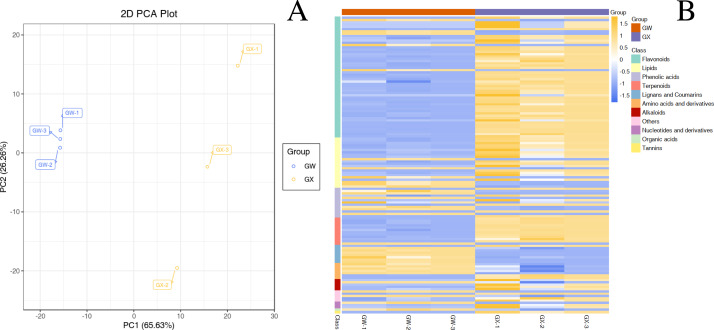
Differential chemotype metabolites between ‘GW’ and ‘GX’. The numbers 1, 2, and 3 indicate three biological replicates. (A) PCA analysis of metabolites identified from ‘GW’ and ‘GX’ (B) Cluster analysis of metabolites from samples of ‘GW’ and ‘GX’. The colour indicates the level of accumulation of each metabolite, from low (green) to high (red).

In total, 477 metabolites were analyzed through PCA analysis (S1). The first principal component (PC1) accounted for 65.63% of the variance, whereas the second principal component (PC2) accounted for 26.26% of the variance (Fig. 2A), indicating a significant difference between GW and GX. The heatmap of all metabolites was created to visualize the variations between GW and GX (Fig. 2B). PCA and the heatmap reveal the significant variations in metabolites between the two developmental stages.

**Figure 3:**
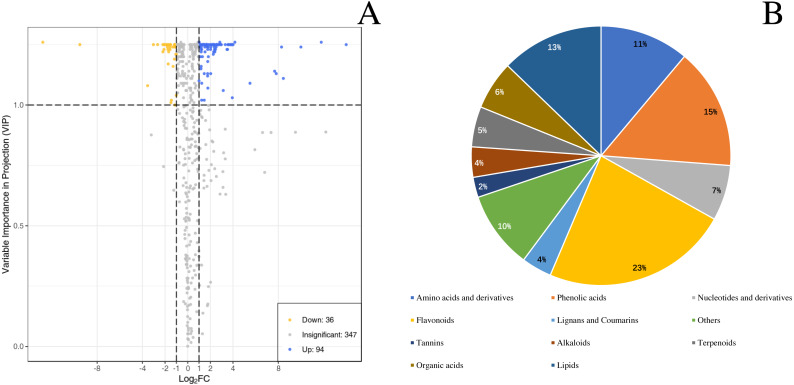
Differential metabolites between ‘GW’ and ‘GX’. (A) Volcano plot of the 124 metabolites identified. (B) Pie chart depicting the categories of the differential metabolites identified between ‘GW’ and ‘GX’.

By identifying the differential metabolites between GX and GW, it can be found out that metabolites with fold change ≥2 or ≤0.5 and the VIP values greater than 1. There are a total of 130 differentiated metabolites, of which 94 are up-regulated and 36 are down-regulated (Fig. 3A). The 130 metabolites can be divided into 11 different categories, with phenolic acids, flavonoids, terpenoids, and lipids in the majority (Fig. 3B).

### Differential metabolite KEGG classification and enrichment analysis

**Figure 4:**
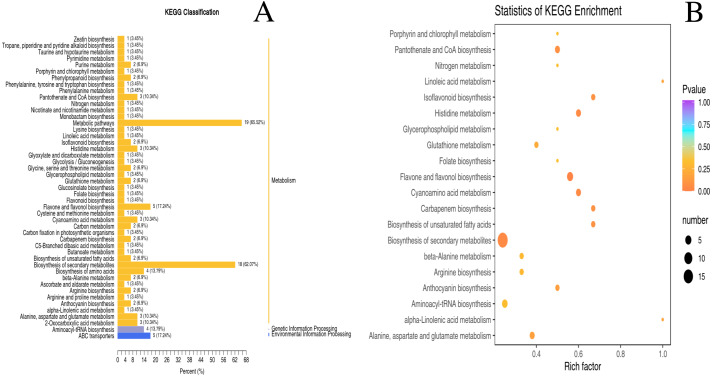
KEGG enrichment map of differential metabolites form ‘GW’ and ‘GX’. (A) KEGG differential enrichment classification map. The *x* axis indicates the proportion and number of metabolites annotated to the pathway, and the *y* axis indicates name of the KEGG metabolic pathway; (B) Statistics of KEGG enrichment. The *x* axis indicates the rich factor corresponding to each pathway, and the *y* axis indicates name of the KEGG metabolic pathway. The color of the point represents the *p*-values of the enrichment analysis. The size and color of bubbles represent the number and degree of enrichment of different metabolites.

The metabolic pathways of 130 differential metabolites were mapped to the KEGG database (S2). Despite few metabolites classified as genetic information processing and environmental information processing, the majority of metabolites were categorized into ‘metabolism’ (Fig. 4A). As revealed by the enrichment analysis of biological pathways associated with two nutrient metabolisms (“pantothenate and CoA biosynthesis” and “flavone and flavonol biosynthesis”), there were significant differences between unripe and ripe *D. delavayi*: *p* < 0.05 (Fig. 4B).

### Amino acids and derivatives, phenolic acids, organic acids

**Figure 5:**
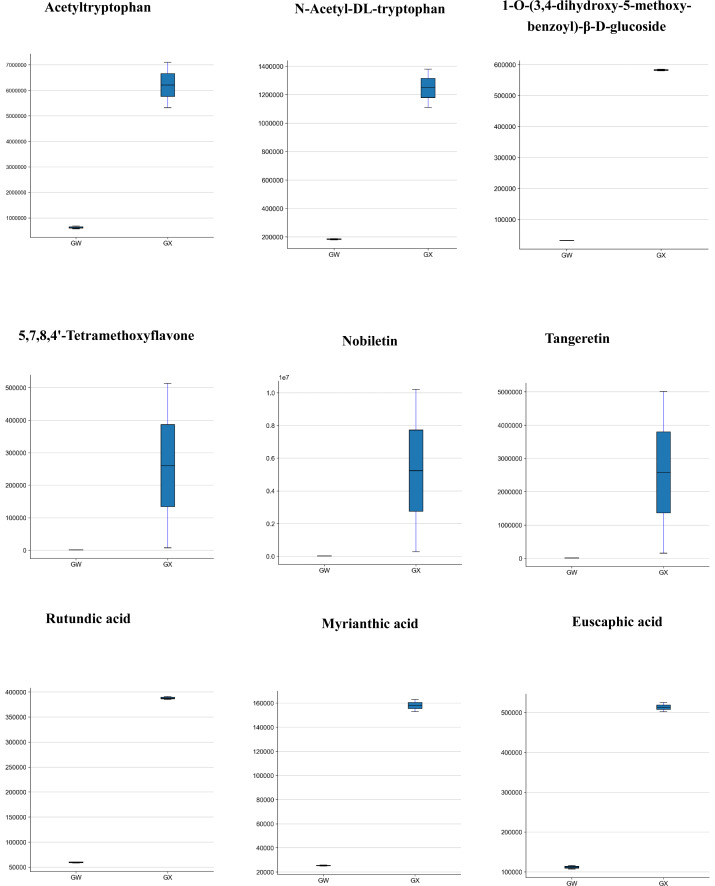
Differences in the contents of 9 metabolites in GW (left) and GX (right).

Concerning the types of metabolites that may affect the flavour of *D. delavayi*, both ‘GW’ and ‘GX’ contained amino acids and their derivatives, with L-glutamic acid and L-phenylalanine in the majority. According to fold change and VIP values, seven differential metabolites were selected. The concentrations of acetyltryptophan and n-acetyl-dl-tryptophan were significantly higher in ‘GX’, whereas the content of other five amino acids in ‘GW’ was lower than in ‘GX’ (Fig. 5), despite no significant change (Table 2).

**Table 2:** Statistics of differentially accumulating amino acids and derivatives, phenolic acids, organic acids in the ‘GW and ‘GX’.

Class	Compounds	Peak area		Fold change	VIP( ≥1)	Type
		GW	GX			
Amino acids and derivatives	L-Asparagine	1,830,000 ± 1,370,000	260,000 ± 14,600	0.086	1.08	down
	L-AsparticAcid	1,120,000 ± 1,040,000	763,000 ± 313,000	0.498	1.04	down
	L-Glutamic acid	19,500,000 ± 17,500,000	9,930,000 ± 5,220,000	0.409	1.16	down
	L-Phenylalanine	5,100,000 ± 4,790,000	2,880,000 ± 652,000	0.358	1.01	down
	1-Methylhistidine	124,000 ± 119,000	41,400 ± 29,100	0.290	1.24	down
	Acetyltryptophan	679,000 ± 568,000	7,100,000 ± 5,320,000	9.957	1.25	up
	N-Acetyl-DL-tryptophan	188,000 ± 179,000	1,380,000 ± 1,110,000	6.800	1.25	up
Phenolic acids	4-Aminobenzoic acid	222,000 ± 222,000	736,000 ± 677,000	3.183	1.25	up
	3,4-Dihydroxybenzaldehyde	69,600 ± 42,900	18,300 ± 16,800	0.312	1.22	down
	Vanillin	998,000 ± 859,000	278,000 ± 271,000	0.296	1.25	down
	Methyl-(2,4-dihydroxyphenyl) acetate	5,560 ± 3,810	11,000 ± 9,190	2.156	1.20	up
	Sinapinaldehyde	542,000 ± 494,000	124,000 ± 120,000	0.236	1.25	down
	Salicin	10,400 ± 4,590	4,310 ± 2,480	0.453	1.00	down
	5-(2-Hydroxyethyl)-2-O-glucosylohenol	304,000 ± 272,000	760,000 ± 735,000	2.596	1.25	up
	1-O-Galloyl- *β*-D-glucose	3,250,000 ± 2,900,000	1,540,000 ± 1,350,000	0.470	1.24	down
	3-O-Caffeoylshikimic acid	173,000 ± 159,000	78,200 ± 69,500	0.445	1.25	down
	1-O-(3,4-dihydroxy-5-methoxy-benzoyl)- *β*-D-glucoside	32,400 ± 32,100	585,000 ± 580,000	18.048	1.26	up
	Glucosyringic Acid	2,640,000 ± 2,510,000	1,180,000 ± 1,130,000	0.448	1.25	down
	Quillaic acid	288,000 ± 270,000	2,220,000 ± 1,980,000	7.527	1.25	up
Organic acids	Phosphoenolpyruvic acid	101,000 ± 87,200	52,600 ± 38,100	0.482	1.21	down

A total of 72 phenolic acids were identified in ‘GW’ and ‘GX’ (Table 1), of which 12 exhibited significant differences in accumulation. 1-O-(3,4-dihydroxy-5-methoxy-benzoyl)-*β*-D-glucoside accumulated in significantly higher concentrations in ‘GX’ than in ‘GW’ (Fig. 5). However, only one of the 29 organic acids was identified as a differential metabolite (phosphoenolpyruvic acid), with ‘GX’ showing a considerably higher cumulative concentration of phosphoenolpyruvic acid than ‘GW’ (Table 2).

### Flavonoids and terpenoids

What is noteworthy is the primary source of nutrients in *D. delavayi,* in addition to the metabolites. Flavonoids were found to have the largest number of unique metabolites. In GW and GX, only four flavonoids were down-regulated, while the remaining 47 flavonoids were up-regulated to varying degrees (Table 3). The three flavonoids that were up-regulated to the most significant extent include 5,7,8,4′-tetramethoxyflavone (348.620 fold), nobiletin (226.216 fold), and tangeretin (204.974 fold) (Fig. 5). These three differential metabolites were all associated with nobiletin and tangeretin and were detected to have higher concentrations in ‘GX’ than in ‘GW. ‘GX’ contained a substantially larger amount of flavonoids than GW.

**Table 3:** Difference of flavonoids in ‘GW and ‘GX.

Class	Compounds	Peak area		Fold Change	VIP (≥1)	Type
		GW	GX			
Dihydroflavone	Liquiritin	704,000 ± 638,000	253,000 ± 210,000	0.345	1.25	down
Dihydroflavonol	Dihydroquercetin (Taxifolin)	193,000 ± 162,000	50,700 ± 32,300	0.234	1.23	down
	Hesperetin 5-O-glucoside	458,000 ± 424,000	969,000 ± 903,000	2.122	1.25	up
Anthocyanins	Rosinidin O-hexoside	57,200 ± 43,900	18,500 ± 15,800	0.339	1.24	down
	Cyanidin-3-O-(6″-p-Coumaroylglucoside)	267,000 ± 219,000	1,120,000 ± 997,000	4.358	1.25	up
	Delphinidin-3-O- (6″-p-Coumaroylglucoside)	1,470,000 ± 1,050,000	6,620,000 ± 6,130,000	5.058	1.25	up
Flavonoid	5,7,8,4′-Tetramethoxyflavone	760 ± 733	513,000 ± 7,560	348.620	1.11	up
	Nobiletin	28,300 ± 18,000	10,200,000 ± 282,000	226.216	1.13	up
	Kaempferol-3-arabinopyranoside	151,000 ± 143,000	385,000 ± 381,000	2.605	1.25	up
	Pinocembrin 7-O- *β*-D-glucoside (Pinocembroside)	171,000 ± 158,000	59,900 ± 56,100	0.352	1.25	down
	Luteolin-4′-O- *β*-D-glucoside*	2,750,000 ± 2,470,000	7,720,000 ± 6,430,000	2.711	1.25	up
	Isoscoparin	132,000 ± 109,000	407,000 ± 367,000	3.216	1.25	up
	Chrysoeriol-5-O-hexoside	3,900,000 ± 3,810,000	12,700,000 ± 11,600,000	3.146	1.25	up
	Taxifolin-3′-O- *β*-D-glucoside	1,110,000 ± 1,090,000	2,500,000 ± 2,280,000	2.173	1.25	up
	Chrysoeriol-O-acetylhexoside	348,000 ± 335,000	1,350,000 ± 1,320,000	3.906	1.25	up
	Isorhamnetin O-malonylglucoside	124,000 ± 109,000	294,000 ± 242,000	2.304	1.24	up
	Kaempferol-7-O-neohesperidoside	106,000 ± 104,000	513,000 ± 489,000	4.771	1.25	up
	Quercetin-3-O- *β*-D-xylopyranosyl- (1 →2)- *β*-D-galactopyranoside	63,300 ± 47,400	342,000 ± 198,000	4.880	1.22	up
	Quercetin 3-O-(6″-trans-p-Coumaroyl)- *β*-D-galactopyranoside	6,460 ± 6,250	46,200 ± 44,600	7.142	1.26	up
	Luteolin-8-C-hexosyl-O-hexoside	81,700 ± 67,100	388,000 ± 379,000	5.157	1.25	up
	Kaempferol-3,7-di-O- *β*-D-glucopyranoside	24,100 ± 17,500	124,000 ± 88,500	5.104	1.24	up
Flavonols	Tangeretin	15,000 ± 10,200	5,010,000 ± 158,000	204.974	1.14	up
	Kaempferol-7-O-rhamnoside	32,000 ± 29,400	70,900 ± 60,100	2.134	1.24	up
	Kaempferol-3-O-rhamnoside (Afzelin)(Kaempferin)	50,200 ± 37,000	108,000 ± 90,600	2.276	1.22	up
	3,5,6,7,8,3′,4′-Heptamethoxyflavone	2,930 ± 2,610	239,000 ± 11,100	45.138	1.09	up
	Kaempferol-3-O-glucoside (Astragalin)*	1,720,000 ± 1,670,000	5,060,000 ± 4,950,000	2.951	1.25	up
	Kaempferol-3-O- *β*-D-glucuronide	26,200 ± 21,900	152,000 ± 78,400	4.791	1.21	up
	Quercetin-3-O- *β*- D-glucoside(Isoquercitrin)*	3,800,000 ± 3,410,000	9,690,000 ± 7,230,000	2.348	1.23	up
	Quercetin-3-O- *β*-D-Galactoside (Hyperin)*	3,790,000 ± 3,390,000	8,000,000 ± 7,530,000	2.163	1.25	up
	Myricetin 3-O-galactoside	16,300 ± 6,940	138,000 ± 129,000	11.481	1.23	up
	Kaempferol-3-O-(6″-acetyl)-glucoside	742,000 ± 579,000	3,200,000 ± 3,170,000	4.826	1.25	up
	Quercetin-3-O-(6″-O-acetyl)-galactoside	4,270,000 ± 2,500,000	20,300,000 ± 16,800,000	5.472	1.23	up
	Quercetin-3-O-(2″-acetyl)- *β*-D-glucuronide	26,000 ± 13,400	42,100 ± 36,900	2.005	1.10	up
	Kaempferol-3-O-(6″-malonyl)-glucoside	258,000 ± 230,000	888,000 ± 846,000	3.553	1.25	up
	Quercetin-7-O-(6′-O-malonyl)- *β*-D-glucoside	128,000 ± 118,000	424,000 ± 366,000	3.211	1.25	up
	Quercetin-3-O-(6″-O-malonyl)-glucoside	71,700 ± 55,500	375,000 ± 368,000	5.839	1.25	up
	”Myricetin-3-O-(6″-malony)glucoside”	3,140 ± 2,530	40,100 ± 27,700	11.951	1.25	up
	Tiliroside	5,490 ± 4,820	148,000 ± 10,500	15.382	1.03	up
	Kaempferol-3-O-rutinoside(Nicotiflorin)	570,000 ± 526,000	2,400,000 ± 2,150,000	4.148	1.25	up
	Quercetin-3-O-rutinoside (Rutin)	3,760,000 ± 3,110,000	17,200,000 ± 15,500,000	4.753	1.25	up
	6-Hydroxykaempferol-7,6-O-Diglucoside	82,700 ± 74,700	622,000 ± 614,000	7.853	1.25	up
	6-Hydroxykaempferol-3,6-O-Diglucoside	48,900 ± 46,500	289,000 ± 273,000	5.891	1.25	up
	Quercetin-5-O-malonylhexosyl-hexoside	18,800 ± 16,700	274,000 ± 269,000	15.272	1.25	up
	Quercetin-7-O-malonylhexosyl-hexoside	27,600 ± 20,200	157,000 ± 116,000	5.704	1.24	up
	Quercetin-O-rutinoside-hexose	9,150 ± 6,340	133,000 ± 93,900	14.669	1.25	up
Flavonoid carbonoside	Isohemiphloin	28,700 ± 21,500	74,200 ± 49,500	2.463	1.20	up
	8-C-Hexosyl-hesperetin O-hexoside	20,000 ± 15,700	213,000 ± 208,000	11.794	1.25	up
Flavanols	Gallocatechin-Gallocatechin	21,300 ± 17,800	113,000 ± 113,000	5.775	1.25	up
Isoflavones	Calycosin	62,100 ± 59,700	292,000 ± 208,000	4.105	1.24	up
	Sissotrin	1,930 ± 1,350	25,700 ± 19,400	13.740	1.25	up
	2′-Hydoxy,5-methoxy Genistein-O-rhamnosyl-glucoside	43,200 ± 37,900	131,000 ± 60,100	2.357	1.09	up

Terpenoids are also the important metabolites contained in *D. delavayi* fruit, as revealed in this study. A majority of terpenoids found to be clearly different between ‘GW’ and ‘GX’ were triterpenes (Table 4). In GW, only one triterpene (3-o-trans-feruloyl euscaphic acid) was down-regulated, while the others were up-regulated. The three triterpenes that were up-regulated most significantly include rutundic acid (6.539 fold), myrianthic acid (6.245 fold) and euscaphic acid (4.597 fold) (Fig. 5). These findings indicate an increase in flavonoids and terpenoids.

**Table 4:** Difference of terpenoids (triterpene) in ‘GW and ‘GX.

Class	Compounds	Peak area		Fold change	VIP( ≥1)	Type
		GW	GX			
Terpenoids (Triterpene)	Sanguisorbigenin	41,300 ± 41,200	92,000 ± 84,200	2.135	1.25	up
	2,3-Dihydroxy 5(6),12(13) diene ursolic acid	159,000 ± 136,000	363,000 ± 346,000	2.400	1.25	up
	Camaldulenic acid	3,360,000 ± 2,870,000	8,010,000 ± 7,680,000	2.518	1.25	up
	3 *β*,19 *α*- Dihydroxyolean-12-en-28-oic acid	27,500 ± 25,500	54,800 ± 52,900	2.031	1.25	up
	Rosamultic acid	1,510,000 ± 1,500,000	3,060,000 ± 3,050,000	2.031	1.26	up
	Ursolic acid-OCH3	2,030,000 ± 1,550,000	4,200,000 ± 4,000,000	2.291	1.23	up
	Rutundic acid	60,600 ± 58,100	391,000 ± 385,000	6.539	1.25	up
	Euscaphic acid	116,000 ± 107,000	525,000 ± 502,000	4.597	1.25	up
	Arjunic Acid	191,000 ± 167,000	541,000 ± 477,000	2.844	1.25	up
	1 *β*,2 *α*,3 *α*,19 *α*-Tetrahydroxyurs-12 -en-28-oic acid	15,800 ± 15,500	79,600 ± 47,300	4.060	1.23	up
	Myrianthic acid	25,700 ± 24,900	163,000 ± 153,000	6.245	1.25	up
	3-O-Trans-feruloyl euscaphic acid	51,700 ± 50,300	6,630 ± 5,880	0.123	1.25	down

## Discussion

So far, the widely targeted metabolite identification analysis based on UPLC-MS/MS has been conducted for the large-scale metabolite identification of numerous plant species (Oikawa et al., 2015; Wang et al., 2017). The existing research of *D. delavayi* focuses mainly on only a few metabolites, such as the determination of tannins (Tiep et al., 2018), the extraction of flavonoids (Deng et al., 2014), and the validation of the biological activity (Mao, Li & Li, 2006). In the present study, the UPLC-MS/MS technique was applied to understand the metabolomic differences between unripe *D. delavayi* and ripe *D. delavayi* and to conduct the most comprehensive survey of fruit metabolites. Finally, a total of 477 metabolites were identified, among which there were 130 distinct metabolites between unripe and ripe fruit. The data provide a comprehensive analysis of metabolic changes between unripe *D. delavayi* and ripe *D. delavayi*. This lays a foundation for the metabolic studies of fruit quality. More importantly, the findings of this study provide reference for fruit tree breeding and contribute new ideas to the planting of high-quality fruit.

Herein, the organic acid content was found lower in unripe *D. delavayi* than in ripe *D. delavayi*. This is likely attributable to the fact that most fruits require the consumption of organic acids as a respiratory substrate during ripening, which provides sufficient energy for fruit development (Merewitz et al., 2012; Sui et al., 2017). Notably, the sugar content rises as the fruit matures (Zhao et al., 2016). This phenomenon is reflected as the preferential conversion of starch by external photoassimilates into sugar (Qiu et al., 2020). However, the sugar content remained basically unchanged in ripe and unripe *D. delavayi*, which is most likely due to *D. delavayi* being distributed in the low-altitude arid regions in southwest Yunnan (Zhao et al., 2012), and the inhibited sugar synthesis by drought stress during fruit development (Wang et al., 2018). However, a further research is required to reveal the metabolic sugar mechanism of *D. delavayi*.

In comparison to other Rosaceae fruits, *D. delavayi* has a higher tannin content (Ben Salem et al., 2017). Tannins are considered to produce anti-inflammatory and antioxidant effects (Parveen et al., 2018), which enhances the anti-inflammatory effects of *D. delavayi* (Xukun et al., 2014). Therefore, it is applicable as a fruit supplement to improve human health. The pattern of change in amino acid content in *D. delavayi* is similar to tomato and Loquat (Xu et al., 2019; Polak et al., 2021). In addition, ripe fruit contains more amino acids than unripe fruit, which is likely due to greater maturity and longer daylight hours (Snowden et al., 2015; Zou et al., 2020).

According to our findings, *D. delavayi* contains plenty of flavonoids and triterpenoids, of which the flavonoids related to nobiletin and tangeretin is most significantly up-regulated. Prior research has demonstrated that these compounds contribute significantly to the colour change of fruit pericarp (Wang et al., 2020; Zhang et al., 2020). Therefore, it is hypothesized in this study that this is one of the reasons why ripe *D. delavayi* is red or yellow. During the ripening of *D. delavayi*, the triterpenoids exhibit a similar upward trend to jujube triterpenoids (Song et al., 2020), as discovered in this study. Triterpenoids promote the biosynthesis of the fruit cuticle, which thickens the cuticle during ripening (Snowden et al., 2015). This enhances the protection against biotic and abiotic stresses during ripening, which ensures the integrity of fruit during development (Tafolla-Arellano et al., 2017; Li et al., 2020).

KEGG enrichment analysis was conducted to reveal the most significant differences between ripe and unripe metabolites from the phenolic pathway (flavonoid biosynthesis and flavonol biosynthesis) (GX and GW). A vast majority of the up-regulated compounds as enriched in these pathways have antioxidative and anti-inflammatory properties (e.g., isoquercitrin and astragalin) (Ferrer et al., 2008), which is consistent with the result of prior research on the medicinal quality of *D. delavayi*. Meanwhile, this provides a reference for future research on the physiology of *D. delavayi* during development.

## Conclusion

Herein, the metabolome is applied to conduct a comprehensive analysis of the metabolic changes between unripe *D. delavayi* and ripe *D. delavayi*, which lays a foundation for the metabolic studies of fruit quality. These distinct metabolites are predominantly involved in the biosynthesis and synthesis of secondary metabolites, such as pantothenic acid, flavonoids, and amino acids. Notably, these metabolites are closely related to the medicinal properties of *D. delavayi*. The findings of the study shed new light on the breeding of fruit trees and the planting of high-quality fruits.

##  Supplemental Information

10.7717/peerj.14441/supp-1 Supplemental Information 1 All metabolites of the fruit of Dravai

10.7717/peerj.14441/supp-2 Supplemental Information 2 Flavonoid biosynthesis and flavonol biosynthesis pathway in D. delavayi fruit (comparison between GW and GX)

10.7717/peerj.14441/supp-3 Supplemental Information 3 The correlation plot

10.7717/peerj.14441/supp-4 Supplemental Information 4 Differential metabolites Pathway KEGG

## Additional Information and Declarations

### Field Study Permissions

The following information was supplied relating to field study approvals (i.e., approving body and any reference numbers): The samples for this study are common and the collection of samples will not cause damage to the environment. The samples were collected with the consent of Liu Yu and Zhang Xinluo villagers; Nuozadu village committee for supporting this study.

### Data Availability

The following information was supplied regarding data availability: The raw data is available in the Supplementary Files.
