# Supplementary figures and images for: Identification of nutritional components in unripe and ripe Docynia delavayi (Franch.) Schneid fruit by widely targeted metabolomics
Source: PeerJ. 2022 Dec 12;10:e14441. doi: 10.7717/peerj.14441 (PMC9753743; doi:10.7717/peerj.14441)

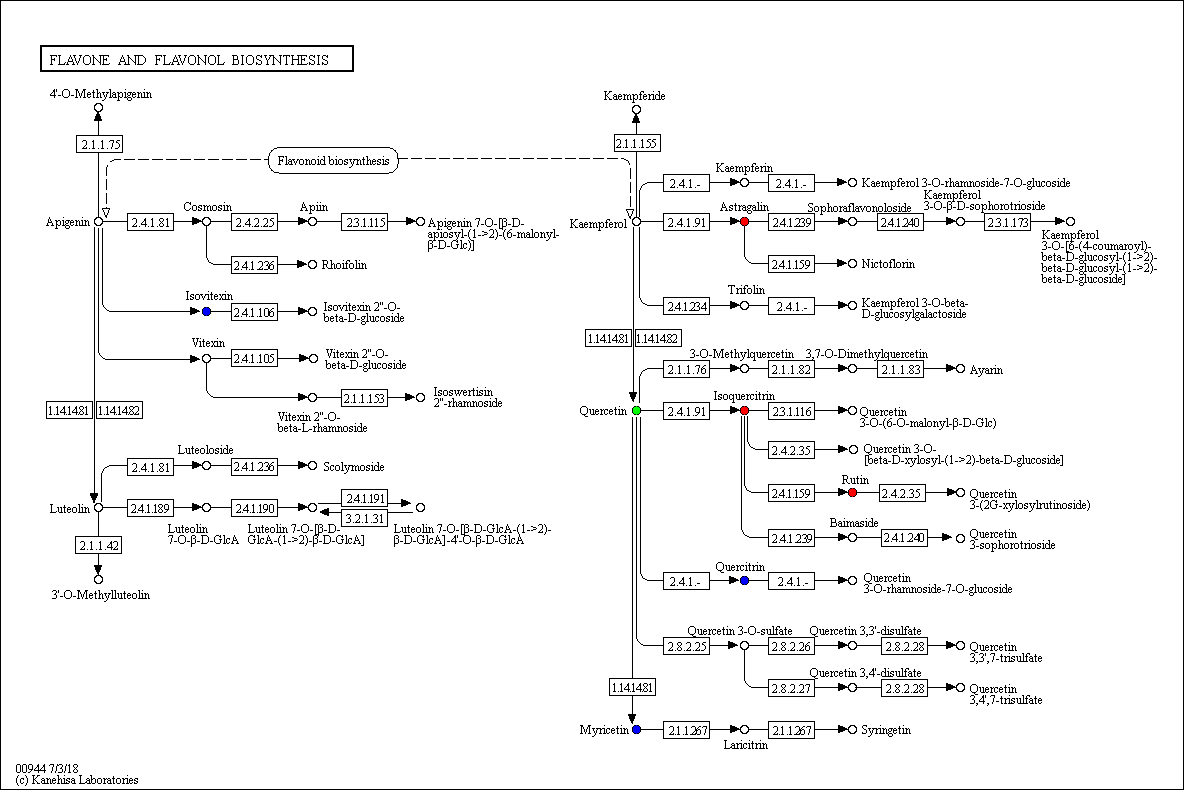

Supplement: Supplemental Information 2 [file peerj-10-14441-s002.png]

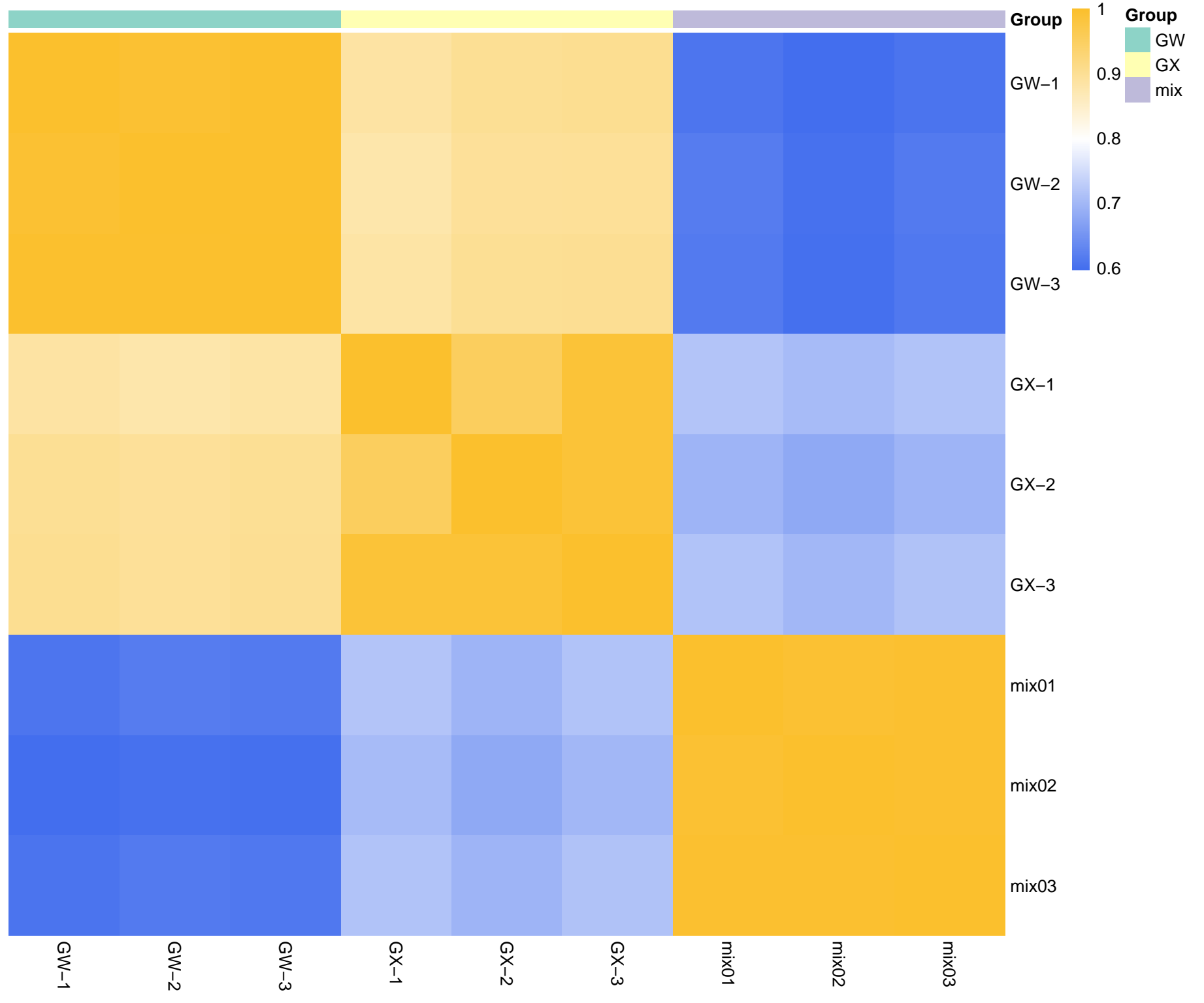

Supplement: Supplemental Information 3 [file peerj-10-14441-s003.pdf]
